# Auditory and Non-Auditory Contributions for Unaided Speech Recognition in Noise as a Function of Hearing Aid Use

**DOI:** 10.3389/fpsyg.2017.00219

**Published:** 2017-02-21

**Authors:** Anja Gieseler, Maike A. S. Tahden, Christiane M. Thiel, Kirsten C. Wagener, Markus Meis, Hans Colonius

**Affiliations:** ^1^Cluster of Excellence ‘Hearing4all’, University of OldenburgOldenburg, Germany; ^2^Cognitive Psychology Lab, Department of Psychology, University of OldenburgOldenburg, Germany; ^3^Biological Psychology Lab, Department of Psychology, University of OldenburgOldenburg, Germany; ^4^Hörzentrum Oldenburg GmbHOldenburg, Germany

**Keywords:** speech reception threshold, hearing impairment, aging, cognition, verbal intelligence, loudness scaling, audiogram profile

## Abstract

Differences in understanding speech in noise among hearing-impaired individuals cannot be explained entirely by hearing thresholds alone, suggesting the contribution of other factors beyond standard auditory ones as derived from the audiogram. This paper reports two analyses addressing individual differences in the explanation of unaided speech-in-noise performance among *n* = 438 elderly hearing-impaired listeners (*mean* = 71.1 ± 5.8 years). The main analysis was designed to identify clinically relevant auditory and non-auditory measures for speech-in-noise prediction using auditory (audiogram, categorical loudness scaling) and cognitive tests (verbal-intelligence test, screening test of dementia), as well as questionnaires assessing various self-reported measures (health status, socio-economic status, and subjective hearing problems). Using stepwise linear regression analysis, 62% of the variance in unaided speech-in-noise performance was explained, with measures *Pure-tone average (PTA), Age*, and *Verbal intelligence* emerging as the three most important predictors. In the complementary analysis, those individuals with the same hearing loss profile were separated into hearing aid users (HAU) and non-users (NU), and were then compared regarding potential differences in the test measures and in explaining unaided speech-in-noise recognition. The groupwise comparisons revealed significant differences in auditory measures and self-reported subjective hearing problems, while no differences in the cognitive domain were found. Furthermore, groupwise regression analyses revealed that *Verbal intelligence* had a predictive value in both groups, whereas *Age* and *PTA* only emerged significant in the group of hearing aid NU.

## Introduction

Among elderly listeners, hearing impairment is typically associated with increased difficulties in understanding speech, especially in adverse listening conditions such as the presence of noise (CHABA, 1988). Speech recognition in noise is commonly assessed by means of speech reception thresholds (SRT) (Plomp, 1986) indicating the signal-to-noise ratio necessary to understand 50% of the presented speech in noise. It is generally recognized that hearing-impaired individuals show inferior speech recognition compared to normal-hearing listeners, yet, hearing thresholds alone cannot fully account for these differences. While predictions based on audibility have been found to be mostly accurate in quiet conditions, they overrate the performance of elderly listeners in noise (Schum et al., 1991; Hargus and Gordon-Salant, 1995; Sommers and Danielson, 1999). Consequently, other factors besides audiometric hearing thresholds seem to contribute to speech-in-noise recognition, which might be likewise affected by supra-threshold and central auditory processing (e.g., Anderson et al., 2013a,b; Füllgrabe et al., 2015) and furthermore, non-auditory processes such as cognition (e.g., Humes, 2005; Wingfield and Tun, 2007).

A number of studies have focused on the contribution of cognitive abilities, confirming a link between certain cognitive abilities and speech recognition in noise in older hearing-impaired listeners (e.g., Gatehouse et al., 2003; Lunner, 2003; George et al., 2007; Lunner and Sundewall-Thorén, 2007; Humes et al., 2013). When accounting for effects of hearing loss by restoring audibility (e.g., via spectral shaping), age and cognitive factors accounted for as much as 30–50% of the variance in speech-recognition performance of elderly hearing-impaired listeners (Humes, 2007). The strength of this relationship varies and seems to depend on the cognitive components addressed as well as the specific tests used. A review by Akeroyd (2008) found that none of the cognitive components hitherto investigated always yielded significant results. Accordingly, measures of general ability, such as IQ, were mostly found as unsuccessful predictors. In contrast, measures of working memory capacity were found most consistently predictive for the speech-in-noise performance of older hearing-impaired listeners, particularly when assessed with the Reading Span Test (Daneman and Carpenter, 1980). Rudner et al. (2011) confirmed the link to speech-in-noise performance by showing that working memory is a relevant factor in both unaided and aided listening conditions in hearing-impaired older listeners. While there is evidence that this link might also exist in normal-hearing older listeners (Füllgrabe and Rosen, 2016a), a recent meta-analysis by Füllgrabe and Rosen (2016b) cautions against a generalization of the link between working memory and speech-in-noise independent of age and hearing loss, as no evidence for such was found for younger normal-hearing listeners. Moreover, the relationship between cognitive capacities and speech recognition has been investigated in the context of hearing aid processing algorithms (e.g., Gatehouse et al., 2003; Lunner, 2003; Souza et al., 2015) and extended to inter-individual differences in the benefit obtained from a hearing aid (Humes, 2003; Ng et al., 2013; Meister et al., 2015).

Besides hearing thresholds and cognitive factors, further auditory measures have been linked to speech intelligibility such as measures of spectral and temporal resolution (e.g., Noordhoek et al., 2001; George et al., 2006, 2007), temporal envelope and temporal fine structure (e.g., Grose and Mamo, 2010; Moore et al., 2012; Füllgrabe, 2013; Füllgrabe et al., 2015; Wallaert et al., 2016) as well as loudness recruitment (Villchur, 1974; Moore and Glasberg, 1993; Van Esch and Dreschler, 2015). Apparently, all of the aforementioned factors relate to speech intelligibility in noise, although to varying degrees. Overall, age and hearing thresholds as measured by the pure-tone audiogram have consistently been found as good predictors across various studies (van Rooij and Plomp, 1990, 1992; Humes et al., 1994; Humes, 2002; for review see Akeroyd, 2008; Houtgast and Festen, 2008). Still, there remains unexplained variance in the prediction of speech-in-noise performance. Houtgast and Festen (2008) summarized that the predictor variables considered up to that point, comprising different auditory and cognitive measures as well as age, fail to fully explain the variability in speech-in-noise recognition with about 30% being unaccounted for, and conclude that not all components relevant for explaining individual differences in speech recognition have been discovered so far. This raises the question which other components might be of additional predictive value.

Despite the technological advances in assistive hearing devices, studies from high-income countries show that hearing impairment remains undertreated (Fischer et al., 2011; Chien and Lin, 2012), particularly among those with mild levels of hearing loss. Accordingly, 40% of moderately hearing-impaired individuals were using hearing aids in the US, but less than 4% of those with a mild hearing impairment (Lin et al., 2011; Chien and Lin, 2012). Moreover, there is a gap of more than 10 years between initial diagnosis and first hearing aid supply (Davis et al., 2007). This is of particular relevance since an uncorrected hearing loss is associated with lower quality of life, reduced social activity, isolation, and increased symptoms of depression (for review see Arlinger, 2003). Arlinger (2003) also reports a number of studies pointing to significant correlations between hearing loss and a decline of cognitive functions giving indirect evidence that hearing aid use may attenuate the effects of cognitive decline, although no causal link was provided. Given the accumulating evidence for an interaction between hearing aid use and various individual factors, it is reasonable to assume that hearing aid supply may have a mediating effect on the prediction of speech-in-noise performance. Previous studies focused on hearing aid users (HAUs) (e.g., Humes, 2002; Rudner et al., 2011), analyzed mixed groups with both HAUs and non-users (NU) (e.g., Humes et al., 2013) or did not clearly state whether the hearing-impaired individuals were using hearing aids. More importantly, none of the studies clearly differentiated between HAU and NU with the same hearing loss profile for predicting unaided speech-in-noise performance.

Summing up, a growing body of evidence suggests that factors beyond standard audiometric measures account for inter-individual differences in speech recognition in noise, although results are equivocal and unexplained variance hints at the potential contribution of further factors or possibly, measurement errors. Many studies used rather time-consuming experimental methods or examined a small number of subjects. Here, we addressed these issues by investigating speech-in-noise recognition of a large sample of hearing-impaired elderly listeners, tested with a battery of different auditory and non-auditory measures. Furthermore, given that up to date a comparison between HAU and NU is missing, we differentiated between older hearing-impaired HAU and NU with the same audiogram profile for groupwise analyses. It is to point out that the test battery was originally designed to extensively test and characterize normal-hearing and hearing-impaired individuals with and without hearing aids out of a cohort, and not primarily with the aim to predict unaided speech-in-noise performance. Thus, measures were selected based on suitability for a clinical setting where measurement time is limited and thus requesting short, easily implementable, yet reliable tests.

Here, two successive analyses are reported, each addressing one aim. (1) The first aim was to identify clinically relevant auditory and non-auditory measures for predicting unaided speech-in-noise recognition for a large sample of older hearing-impaired individuals, and to determine the respective (additional) predictive value for the identified auditory -, cognitive-, self-reported-, and health measures. To that end, a full-data regression was applied on the whole sample of participants using a stepwise regression algorithm (main analysis). (2) Secondly, we compared HAU and NU with the same hearing loss profile regarding potential differences in the test measures and regarding unaided speech recognition to identify whether regressors differ as a function of hearing loss profile and hearing aid use (complementary analysis).

## Main Analysis

### Materials and Methods

#### Participants

**FIGURE 1:**
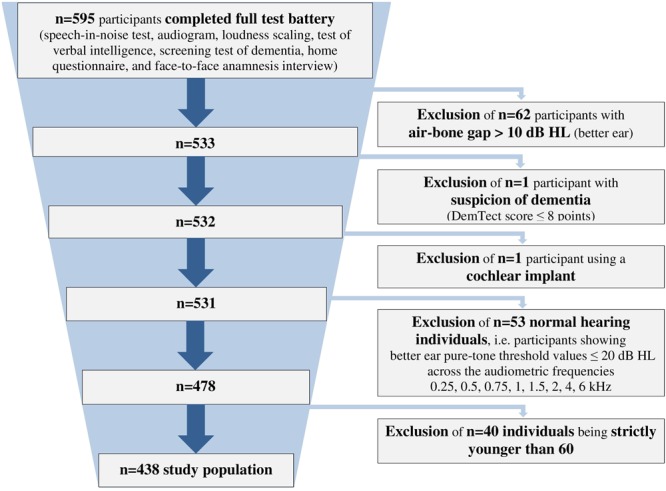
**Flowchart of the study population.** Shown is the application of all exclusion criteria.

We analyzed data from 438 hearing-impaired individuals (166 females, 37.9%; 272 males, 62.1%) with a sensorineural hearing loss aged 60–85 years (*mean* = 71.1 ± 5.8 years). The included participants showed a hearing impairment range from *very mild* to *severe* (Bisgaard et al., 2010), and included both HAU and NU. They were selected from a database with more than 2,400 individuals (Hörzentrum Oldenburg GmbH, Germany) out of whom 595 had completed a test battery comprising auditory and cognitive tests as well as a comprehensive self-report questionnaire. From those, individuals with air-bone gaps > 10 dB HL across the audiometric frequencies 0.5, 1, 2, and 4 kHz, with suspicion of dementia (DemTect scores of ≤8; Kalbe et al., 2004) or those with a cochlear implant were excluded. Furthermore, individuals showing better ear pure-tone threshold values ≤ 20 dB HL across the frequencies 0.25, 0.5, 0.75, 1, 1.5, 2, 4, and 6 kHz were defined as normal-hearing (Thiele et al., 2012) and also excluded. In addition, participants aged younger than 60 years were excluded for the analyses, resulting in the final sample of *n* = 438 (see **Figure 1**).

#### Test Battery

The test battery, which was designed to further characterize an existing cohort, comprised a home questionnaire, and a 1-h test session at the facilities of the Hörzentrum Oldenburg. The test session encompassed an initial face-to-face interview addressing the medical history, a speech-recognition-in-noise test (Goettingen Sentence Test, Kollmeier and Wesselkamp, 1997), two auditory measurements (Audiogram; Adaptive Categorical Loudness Scaling, Brand and Hohmann, 2002), as well as two cognitive tests (Verbal Intelligence, Schmidt and Metzler, 1992; DemTect, Kalbe et al., 2004). All participants were measured unaided, i.e., the HAU group without their hearing aids. The measurements were performed by trained professional staff.

The studies were approved by the local ethics committee of the University of Oldenburg and performed according to the Declaration of Helsinki. All participants gave their written informed consent prior to the study and received a monetary compensation for participation.

##### Auditory measures

For the auditory measures entering the analyses, measures of the better ear were used being defined as the ear with the lower pure-tone average (PTA) of the audiogram’s air conduction measurement across the frequencies 0.5, 1, 2, and 4 kHz. In case of equal PTAs for both sides, the right ear measures were selected. The only exception constituted the Adaptive Categorical Loudness Scaling measures where the average across both ears was used.

*Speech recognition*. Speech reception thresholds in noise were measured with the Goettingen Sentence Test (GOESA) by Kollmeier and Wesselkamp (1997). In GOESA, subjects are presented with three-to-seven-word meaningful sentences one at a time in background noise (goenoise) and instructed to repeat as many of the words as possible. Participants randomly completed 1 of the 10 test lists containing 20 sentences each. Both speech and noise stimuli were presented via a free-field loudspeaker in a sound-attenuated cabin.

The GOESA applied an adaptive SRT measurement (Brand and Kollmeier, 2002) in the corresponding stationary speech-shaped goenoise with a presentation noise level of 65 dB SPL, individually changed to 80 dB SPL for seven participants with a severe hearing impairment. This was applied if the noise at 65 dB was not sufficiently audible (Wagener and Brand, 2005). Starting with a reference level of 0 dB SNR, the background noise remained fixed at 65 or 80 dB, correspondingly, while the target speech varied adaptively to obtain the SRT. Thus, stepping rule, step size, and number of reversals were not fixed but adaptively changed based on the subject’s response (for details see Brand and Kollmeier, 2002). The scores were calculated from the proportion of correctly repeated words which were entered by the experimenter sitting in front of the test subject. Testing of a single list took about 5 min and did not require any prior practice trials.

The *50*%*-SRT*, that is, the signal-to-noise ratio that corresponds to 50% intelligibility, served as the outcome measure for the regression analyses (see Supplementary Table S1)^1^.

*Audiogram*. Pure-tone audiometry was conducted via a Unity II Audiometer with HAD200 headphones: (1) air-conduction hearing thresholds at the frequencies 0.125, 0.25, 0.5, 0.75, 1, 1.5, 2, 4, 6, and 8 kHz, (2) bone-conduction thresholds at the frequencies 0.5, 0.75, 1, 1.5, 2, and 4 kHz, and (3) uncomfortable loudness level at the frequencies 0.5, 1, 2, and 4 kHz. For measuring the uncomfortable loudness level, participants were presented with 1-s pure tones starting at a dB level above threshold and increasing in steps of 5 dB. They are instructed to press a button when their uncomfortable loudness level is reached. The total audiometric testing time was 13 min.

For regression analyses, the *PTA* across the frequencies 0.5, 1, 2, and 4 kHz was submitted as potential predictor variable. Moreover, the *Uncomfortable loudness level (audiogram)* at the single frequencies *0.5, 1, 2*, and *4 kHz* entered the analyses (see Supplementary Table S1).

*Adaptive categorical loudness scaling*. Categorical loudness scaling is a psychoacoustic measurement to assess the individual, subjective loudness perception, especially with respect to hearing deficits, and for the diagnosis of loudness recruitment by obtaining loudness growth functions (e.g., Allen et al., 1990; Hohmann and Kollmeier, 1995). In this study, we applied the Oldenburg-Adaptive Categorical Loudness Scaling (ACALOS) by Brand and Hohmann (2002). Subjects were presented monaurally via HDA200 headphones with test stimuli at different levels. For each stimulus, the participants had to rate their subjectively experienced loudness with stimulus levels presented in pseudorandom order in 20 trials. Measurements were performed successively for 1.5 and 4 kHz narrowband noise stimuli for each ear separately. The average testing time was 8 min.

The 11 categories were assigned values of 0–50 categorical units (CU) in steps of five, from which levels for particular loudness categories can be derived. A monotonically increasing loudness function was fitted to the responses for each of the ACALOS measurements using the BTUX fitting method (Oetting et al., 2014). This resulted in the following variables that can be used to describe the loudness perception and were submitted to the regression analyses: *Hearing threshold level for 1.5* and *4 kHz* (at 2.5 CU), *Medium loudness level for 1.5* and *4 kHz* (at 25 CU), and *Uncomfortable loudness level for 1.5* and *4 kHz* (at 50 CU). Additionally, the slope of the lower part of the fitted loudness function, which is associated with loudness recruitment, entered as potential predictor (*Loudness recruitment for 1.5* and *4 kHz*, see Supplementary Table S1).

##### Cognitive measures

*Verbal intelligence*. Cognitive performance was assessed with the German vocabulary test (Wortschatztest, Schmidt and Metzler, 1992). The test measures verbal intelligence and is an indicator of crystallized intellectual abilities (Schmidt and Metzler, 1992). Moreover, factor analytic validations revealed that the results in vocabulary tests load highly on a g-factor and can thus be regarded as an indicator of general intellectual abilities (g-factor) (Schmidt and Metzler, 1992). In this 7-min test, the participants have to identify the real word among similar non-words. The number of correctly identified words yields the raw score that was used for further analyses (*Verbal intelligence*, see Supplementary Table S1).

*DemTect*. The DemTect test by Kalbe et al. (2004) is a short (8–10 min) and easily administered neuropsychological screening test for dementia. It contains five subtests assessing different cognitive areas, including immediate and delayed memory, working memory, number transcoding and semantic fluency. The subtests comprise the recall of a 10-item wordlist in two trials (“wordlist”), transcoding of numbers in numerals and vice-versa (“number transcoding”), a semantic word fluency task (“verbal fluency”), repeating number sequences in backward order (“digit span reverse”), and the wordlist’s delayed recall (“wordlist delayed recall”). The corresponding variables entering analyses were labeled *Wordlist, Verbal fluency, Number transcoding, Digit reverse*, and *Wordlist delayed recall*, respectively (see Supplementary Table S1).

The transformed, age-normed total score (maximum 18) was used as an exclusion criterion in case of suspected dementia (0–8 points). For regression analyses, the participants’ raw scores of each subtest were used instead of the age-normed scores, as we wanted to relate the inter-individual age-related changes in different cognitive domains to the speech-recognition performance.

##### Self-reports (questionnaire data and anamnesis)

The self-report measures were obtained from a questionnaire being completed at home by the participants and a face-to-face anamnesis interview at the Hörzentrum. The 11-page home questionnaire contained numerous questions regarding the main topics “hearing anamnesis” (e.g., diagnosis and duration of hearing impairment, subjective ratings of hearing problems in quiet and noise, noise exposure, cause of hearing impairment, previous and current hearing aid use, duration of hearing aid use, occurrence of sudden hearing loss, occurrence of middle ear infections, occurrence of hearing impairment in family, questions related to ear noise/tinnitus), “health status” (SF-12 and multimorbidity; see below), “technology commitment” (see below), and “demographic questions” (age, gender). The variables entering the analyses are given in the Supplementary Table S1.

At the beginning of the test session at the Hörzentrum, trained diagnostic staff furthermore performed an anamnesis which encompassed more detailed questions regarding the current hearing aid use as well as questions relating to education, occupation and income with reference to the socio-economic status. Checking the home questionnaire and completing the face-to-face anamnesis took about 13 min.

*Health status: SF-12 and multimorbidity*. The home questionnaire included the German version of the SF-12 Health Survey that constitutes a multipurpose self-reported generic measure of health status and health-related quality of life (Bullinger et al., 1995; Bullinger and Kirchberger, 1998). The SF-12 yields two subscales, a Physical Health Score (PCS) and a Mental Health Score (MCS) which are transformed sum scores such that they range from 0 to 100, with higher scores indicating a better self-reported health status, and standardized to a norm population with *mean* = 50 ± 10 (Bullinger and Kirchberger, 1998). For regression analyses, the transformed and standardized sum scores of the two scales were included as potential predictor variables labeled *Physical sum score* (*SF-12)* and *Mental sum score (SF-12)* (see Supplementary Table S1).

In addition to the self-reported health status derived from SF-12, we obtained an index of multimorbidity for each participant that denotes the concurrent existence of multiple medical conditions in the same person (Scheidt-Nave et al., 2010). The individuals indicated if any of 20 medical conditions had ever been diagnosed by a doctor and whether a diagnosed condition had also occurred within the last 12 months. From the latter, we calculated the multimorbidity score as a summed score of the self-reported conditions present in the past 12 months, which entered as a further predictor variable to the regression analyses (*Multimorbidity sum score*, see Supplementary Table S1).

*Technology commitment*. The questionnaire furthermore contained a short, 12-item scale assessing the technology commitment as this has been found a significant moderator variable for using hearing aids, independent of hearing loss (Neyer et al., 2012). Technology commitment is a composite of the three subscales technology acceptance, technology competence, and technology control (Neyer et al., 2012). For regression analyses, the mean score entered as predictor variable (*Technology commitment*, see Supplementary Table S1).

*Socio-economic status*. The socio-economic status is defined as a measure of one’s combined economic and social status, typically aggregating measures of education, income, and occupation to one index (Baker, 2014). From the face-to-face interview data, we computed an individual socio-economic status score based on the suggestion for a social class index of Winkler and Stolzenberg (2009). Sum scores range from 3 to 21 and were used as a further predictor variable for regression analyses (*Socio-economic status sum score*, see Supplementary Table S1).

### Analysis

In a first step, we aimed to identify auditory and non-auditory measures for predicting speech perception in noise in older hearing-impaired listeners with different degrees of hearing impairment using all 438 participants. We applied a stepwise linear regression algorithm and used the *50*%*-SRT* of the Goettingen sentence test as outcome variable. As potential predictor variables, we selected in a knowledge-driven approach 43 categorical or continuous key measures from the test battery aiming at obtaining a pool of meaningful test measures according to the prediction of speech perception in noise. To avoid high multicollinearity, we only selected representative measures from the tests, subtests, and questionnaire topics. The decision for preselection was based on consensus between the authors. These measures were then grouped and labeled, resulting in six subject areas comprising auditory (13) and demographic (2) measures, self-reports (17), as well as cognitive (6), health (3), and economic-technical (2) measures. Just like the selection of measures, group formation and labeling was done in a knowledge-driven approach with the subject areas not being necessarily mutually exclusive. The potential predictors entering the analyses as well as the outcome variable are shown in the Supplementary Table S1.

**FIGURE 2:**
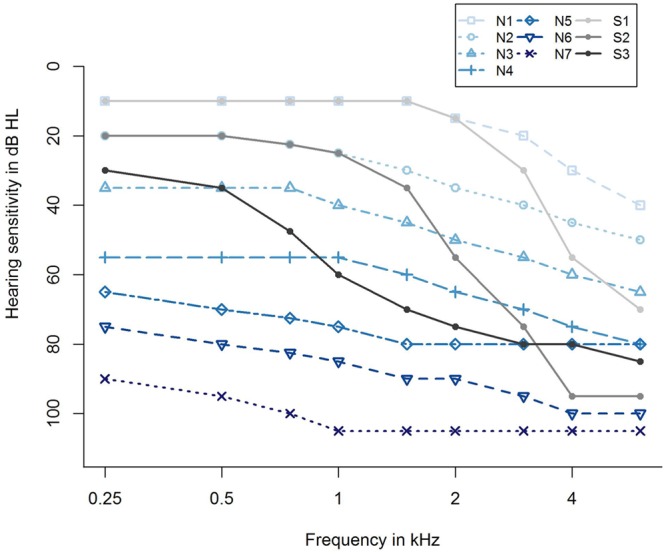
**Ten standard audiograms used for grouping the participants regarding their hearing loss profile (Bisgaard et al., 2010).** Shown are audiogram profiles for individuals with different degrees of hearing loss (N1,…, N7 and S1, S2, S3) distinguishing between a *flat/moderately sloping* (N) and a *steep sloping* hearing loss (S). The profiles N1,…, N7 refer to a *very mild, mild, moderate, moderate/severe, severe, severe*, and *profound* (*flat/moderately sloping*) hearing loss, and S1, S2, S3 indicate a *very mild, mild, and moderate/severe* (*steep sloping*) hearing loss.

For taking into account the different severities of hearing loss, we further categorized the participants with respect to their hearing loss profiles according to Bisgaard et al. (2010). They proposed 10 standard audiograms categorizing hearing impairment from *very mild* to *severe*, and further differentiating between *flat/moderately sloping* and *steep sloping* hearing loss, resulting in seven audiograms for *flat/moderately sloping* hearing loss (N1,…, N7) and three standard audiograms for *steep sloping* hearing loss (S1, S2, and S3), as shown in **Figure 2**. Applying an algorithm by Thiele et al. (2012), the audiogram profile of each participant was assigned to one of the standard audiograms using the method of least squares. This resulted in a grouping variable that was additionally submitted as a potential predictor to the regression analysis, labeled as *Grouping variable (Bisgaard)*, see Supplementary Table S1.

#### Data Pre-processing

2.5% of the data of the potential predictor variables were missing values. The number of missing values per variable is shown in the Supplementary Table S1. Assuming that these values were missing completely at random or missing at random, we imputed those values by applying mean imputation (Wilks, 1932).

#### Stepwise Variable Selection

Linear regression models are commonly compared by means of the Bayesian information criterion (BIC) measuring the trade-off between model fit and model complexity (Schwarz, 1978). Using the BIC-function of the R-package stats, the BIC can be obtained easily for a fitted linear regression model (R Core Team, 2015). For the full-data regression approach, we implemented a stepwise regression algorithm, i.e., a combination of forward selection and backward elimination, called stepBIC for variable selection. StepBIC is a modified version of the stepDIC algorithm described in Tahden et al. (2016), and proceeds as follows: It starts with an empty model containing the outcome variable and an intercept only. Using a pool of potential predictors, the algorithm alternates forward selection and backward elimination steps until it selects the best model according to BIC. The robustness of the implemented stepBIC algorithm was tested by comparing its results with those from a standard stepwise procedure using IBM SPSS Statistics (Version 23).

#### Full-Data Regression

The aim of this study was to identify measures beyond audiological standard measures that influence the *50*%*-SRT* in noise. In a preliminary descriptive step, we calculated two-sided Pearson’s correlation tests (against zero) of the *50*%*-SRT* outcome variable and the potential continuous predictor variables. As shown in Supplementary Table S1, all correlations yielded statistical significance (*p* < 0.01) except for the three health factors (*Physical* and *Mental sum score* of the SF-12, and *Multimorbidity sum score*). Highest positive correlations were obtained between the *50*%*-SRT* and the *PTA* (Pearson correlation coefficient *r* = 0.74) as well as the *Hearing threshold level for 1.5 kHz* of the loudness scaling (*r* = 0.62).

Subsequently, we performed a full-data regression approach entering the *50*%*-SRT* and the 43 potential predictor variables into the stepwise algorithm stepBIC. As the distribution of the *50*%*-SRT* variable was right-skewed, the outcome variable *y* was transformed using the function log(y + 10), with -10 < min(y) < 0, to approximately comply with the normality assumption.

In addition to the pre-processing steps mentioned above, we transformed part of our potential predictors for the data-driven full regression approach: To remove skewness for six of the continuous covariates, a logarithmic, square root or reciprocal transformation was applied (Kuhn and Johnson, 2013). The decision for the respective variable transformation is shown in Supplementary Table S1.

#### Cross-Validation

In order to improve the predictive performance of the full-data regression approach, we applied a resampling technique called *k*-fold cross-validation. This technique uses a subset of the analysis dataset to fit a model and the remaining dataset to estimate the model performance. Here, the analysis dataset consisted of the log-transformed outcome variable, the 43 pre-processed potential predictor variables, and the *Grouping variable (Bisgaard)* of *n* = 438 individuals. Following Kuhn and Johnson (2013), we partitioned the analysis dataset randomly into *k* = 10 subsets with balanced sample sizes. First, the stepwise regression algorithm was applied to part of the analysis dataset comprising all the subsets except the first one (the so-called training set). The resulting model and the held-out subset (the so-called test dataset) were used to estimate the percentage of variance explained and the root-mean-square error. Then, this procedure was reapplied with the second subset as test dataset, and so on. To increase the precision of the estimates, we repeated this 10-fold cross-validation procedure five times, as recommended in Kuhn and Johnson (2013). This resulted in 50 iterations and thus, 50 linear regression models, using 50 different test datasets to estimate model efficacy. Finally, results were summarized and aggregated: The frequencies of predictor selection across the 50 regression models were counted, and the 50 estimated values of the percentage of variance explained as well as the root-mean-square error were averaged.

To strengthen the validity of this analysis, we additionally applied a LASSO regression approach using the glmnet-function of the R-package glmnet (Friedman et al., 2010). We then compared the results with the linear regression analysis.

All analyses were conducted using the statistical SAS software version 9.4 (for data management programs; SAS Institute, 2012) and the software R version 3.2.3 (for analysis programs; R Core Team, 2015).

### Results

In this main analysis, we considered the whole sample of 438 individuals irrespective of degree of hearing impairment and hearing aid use. To characterize these participants regarding their speech-recognition performance in noise, we entered the log-transformed *50*%*-SRT* as outcome variable and the 43 potential predictor variables as well as the grouping factor into the variable selection algorithm stepBIC. The mean percentage of variance explained across the 50 cross-validation iterations was 62.09% and the mean test error (root-mean-square error) was 0.17.

**FIGURE 3:**
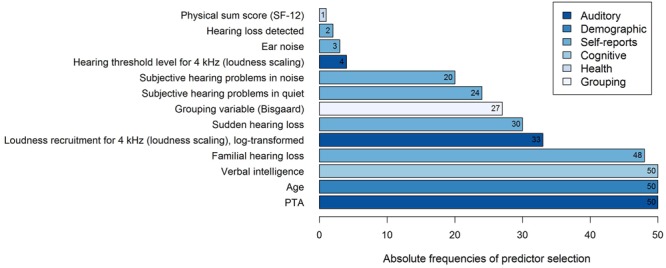
**Frequencies of predictor selection in stepwise regression within a 10-fold cross-validation procedure repeated five times.** Shown are all predictors that were selected by stepBIC across 50 iterations and the absolute frequencies of selection. The selected predictors are ordered by increasing frequencies and the different subject areas of the predictors are mapped in different colors.

Thirteen different variables were selected as predictor variables by the stepwise selection algorithm, as shown in **Figure 3**. In all of the 50 regression models, the three variables *PTA, Age*, and *Verbal intelligence* were selected as explanatory variables for the log-transformed *50*%*-SRT*, followed by the subjectively reported occurrence of *Familial hearing loss* (48 out of 50 iterations).

To examine the predictive performance of the selected variables, we calculated the variance accounted for as the respective variables were added successively to the regression model, as shown in **Table 1**. We therefore ordered the predictors thematically according to their subject areas and considered, in a first and second step, the change in the amount of *R*^2^ by adding the variables *PTA* and *Age* to the regression model, as these variables are known as measures explaining most of the SRT variance (Akeroyd, 2008; Houtgast and Festen, 2008). Then, further auditory measures were added, i.e., the two loudness scaling variables and six auditory self-reported measures. In a fifth step, we entered the cognitive predictor *Verbal intelligence*, subsequently the health variable *Physical sum score (SF-12)*, and finally, the *Grouping variable (Bisgaard)*. Given that the amount of the explained variance depends on the order of the entered predictors described above, the major proportion of the *50*%*-SRT* variance was explained by the *PTA* (58.48%), as presented in **Table 1**. The *Grouping variable (Bisgaard)* added another 2.55%, followed by *Verbal intelligence* with 1.51%. The six self-reports together explained 6.58%, and the variable *Age* 0.96%. The least variance was explained by the further auditory measures (0.52%) and the health measure (0.14%).

**Table 1:** Proportion of variance explained with variable *Age*.

Subject area	Predictor	Δ*R*^2^ (%)
Auditory	*PTA*	58.48

Demographic	*Age*	0.96

Auditory	*Loudness recruitment for 4 kHz (loudness scaling)*,	0.52
	*log-transformed Hearing threshold level for 4 kHz (loudness scaling)*	

Self-reports	*Familial hearing loss*	
	*Sudden hearing loss*	
	*Subjective hearing problems in quiet*	
	*Subjective hearing problems in noise*	6.58
	*Ear noise*	
	*Hearing loss detected*	

Cognitive	*Verbal intelligence*	1.51

Health	*Physical sum score (SF-12)*	0.14

Grouping	*Grouping variable (Bisgaard)*	2.55
		**Total *R*^2^ = 70.74%**

*Shown are all selected predictors in the stepwise regression approach with cross-validation in a thematically summarized and ordered sequence. Furthermore, the corresponding change in the amount of the adjusted coefficient of determination by adding the predictor(s) to the regression model is presented, reflecting the proportion of explained variance.*

*R^*2*^ = adjusted coefficient of determination.*

*ΔR^*2*^ = change in the amount of *R*^*2*^ in % by adding the respective predictor(s) reflecting the proportion of explained variance accounted for as the respective predictor(s) is/are added to the regression model.*

To test whether the inclusion of the variable *Age* might cover the contribution of other variables correlated with it, we performed the same analysis without the variable *Age* as potential predictor. The mean variance explained across the 50 iterations was 60.04% and the mean test error was 0.18. As shown in **Table 2**, the resulting model is similar to the previous one with *PTA* and *Verbal intelligence* again emerging as the most frequently selected predictors (50 out of 50 iterations). In contrast, the auditory measure *Uncomfortable loudness level for 2 kHz (audiogram)* and the cognitive measure *Wordlist (DemTect)* are now selected 15 and one time, correspondingly, while the variables *Ear noise* and *Physical sum score (SF-12)* are no longer among the predictors. While the variance explained by *PTA* remained constant, the predictive value of the other auditory measures increased slightly to 1.19%. The selection of the variable *Wordlist (DemTect)* did not add to the amount of variance accounted for by the cognitive measures compared to the previous model.

**Table 2:** Proportion of variance explained without variable *Age*.

Subject area	Predictor	Δ*R*^2^ (%)
Auditory	*PTA*	58.48

Auditory	*Uncomfortable loudness level for 2 kHz (audiogram)*	1.19
	*Loudness recruitment for 4 kHz (loudness scaling)*,	
	*log-transformed Hearing threshold level for 4 kHz (loudness scaling)*	

Self-reports	*Familial hearing loss*	
	*Sudden hearing loss*	
	*Subjective hearing problems in quiet*	5.99
	*Subjective hearing problems in noise*	
	*Hearing loss detected*	

Cognitive	*Verbal intelligence*	1.51
	*Wordlist (DemTect)*	

Grouping	*Grouping variable (Bisgaard)*	2.09
		**Total *R*^2^ = 69.26%**

*Shown are all selected predictors in the stepwise regression approach with cross-validation in a thematically summarized and ordered sequence.*

*R^*2*^ = adjusted coefficient of determination.*

*Δ*R*^*2*^ = change in the amount of *R*^*2*^ in % by adding the respective predictor(s) reflecting the proportion of explained variance accounted for as the respective predictor(s) is/are added to the regression model.*

Finally, to test the predictive value of *Age* alone, a third analysis was conducted, now only including *Age* as predictor. The proportion of explained variance was 5.66%.

When comparing results of the linear regression approach to LASSO regression, we found that the ten most frequently selected predictors by stepBIC were consistent with the resulting predictors after applying LASSO regression to the analysis dataset.

### Discussion

The main aim of this analysis was to identify clinically relevant auditory and non-auditory measures for predicting unaided speech-in-noise recognition for a large sample of older hearing-impaired individuals, and to determine the respective (additional) predictive value for the identified measures. As the test battery was originally designed to characterize normal-hearing and hearing-impaired individuals while establishing a cohort, clinically relevant refers to the short duration and easy implementation of the corresponding tests in a clinical setting.

The full-data stepwise regression selected 13 out of 43 potential predictor variables accounting for 62% variance in unaided speech-in-noise performance (log-transformed *50*%*-SRT*). This result is in agreement with previous studies typically reporting around 70% explained variance (Houtgast and Festen, 2008), although this study differed from previous ones by a variety of measures of different domains at the same time. In contrast to the reviewed studies, which counted between 10 and 200 hearing-impaired listeners, our large sample of 438 individuals allowed for the inclusion of more potential predictor variables. The additionally performed LASSO regression yielded similar results, supporting the full-data regression approach.

Overall, the variables *PTA* and *Age* were consistently selected as relevant predictors in the full-data regression runs (50 of 50), accounting together for 59% of variance when added successively to a model. This suggests that hearing thresholds contribute substantially to the variance in unaided speech-in-noise recognition for a large sample of older individuals with varying degrees of hearing impairment. Partial correlations between *50*%-*SRT* and *PTA* revealed that the correlation remained almost unchanged when accounting for *Age* (*r* = 0.74, *r*_part_ = 0.73). Moreover, in a second model excluding the variable *Age, PTA* was again consistently selected (50 out of 50). This confirms prior findings identifying hearing loss repeatedly as the primary and best predictor for unaided speech-in noise performance in elderly hearing-impaired listeners (van Rooij and Plomp, 1990, 1992; Humes et al., 1994; Humes, 2002; Akeroyd, 2008; Houtgast and Festen, 2008). The prominent role of the *PTA* reflecting the degree of hearing loss was not unexpected considering the *unaided* measurement of the SRT serving as the outcome variable. The role of the predictor *Age* also conforms to previous studies showing correlations to *SRT* even when accounting for the effects of *PTA*. While *Age* by itself might explain variance in speech-in-noise recognition in a statistical sense, it is rather the factors associated with aging that explain the underlying difficulties in speech-in-noise recognition. The finding that the variable is selected as significant predictor indicates that not all relevant factors for explaining speech-in-noise variability associated with aging might have been identified so far (cf. Houtgast and Festen, 2008), although the additional variance explained by *Age* in our analysis was only 1%. When considering *Age* as the only predictor, merely 6% were accounted for, suggesting that the predictive power of *Age*, although easily and quickly assessed, is not sufficient to replace more time-consuming auditory and cognitive tests for elderly hearing-impaired individuals.

Just like *PTA* and *Age*, the variable *Verbal intelligence* entered the model as significant measure in each of the 50 cross-validations in both models. *Verbal intelligence*, as assessed with a vocabulary test (Wortschatztest), is an indicator of crystallized intellectual abilities and loads highly on a factor of g-factor (Schmidt and Metzler, 1992). The present finding indicates that crystallized intellectual abilities play a role in explaining inter-individual differences in speech-recognition performance such that higher *Verbal intelligence* scores were associated with a better performance, although accounting for only 1.5% of additional variance when added successively after auditory measures and self-reports.

The role of *Verbal intelligence* has to be discussed in the light of previous findings showing a slightly different pattern. While the review of Akeroyd (2008) confirms a link between unaided speech intelligibility in noise and cognitive abilities, although secondary to hearing loss, the overall picture indicated that, in particular, measures of working memory were successful in prediction for older hearing-impaired listeners. In contrast, measures of the general ability such as IQ mostly failed in yielding significant results when examined alone. There are, however, exceptions such as a study by Füllgrabe et al. (2015) who have found a relationship between a composite cognition score and speech-in-noise processing. Furthermore, Humes (2002) derived two factors from the Wechsler Adult Intelligence Scale-Revised, namely an age-independent “Verbal IQ”-factor and a “Non-verbal-IQ and Aging”-factor. Again, hearing loss emerged as primary predictor accounting for most variance, yet, followed by the “Non-verbal-IQ and Aging” and subsequently, the “Verbal IQ”-factor, explaining an additional 7 and 5% variance, respectively. Correspondingly, higher Verbal IQ scores were associated with a better speech-recognition performance which coincides with the present results (cf. also Humes, 2003).

Compared to other cognitive domains important for understanding speech in noise, such as working memory which declines with age (Glisky, 2007), *Verbal intelligence* has been shown to reflect a measure of intellectual ability remaining relatively stable across the lifespan (Schmidt and Metzler, 1992). Accordingly, the degree of correlation between *50*%-*SRT* and *Verbal intelligence* persisted when controlling for *Age* (*r* = -0.25, *r*_part_ = -0.26). Thus, the contribution of *Verbal intelligence* to differences in speech-recognition performance may not be attributed primarily to age-related differences in crystallized intellectual ability. Instead, the present findings suggest that hearing-impaired individuals with higher verbal intellectual abilities perform better in understanding speech in noise, independent of *Age*. When conducting the analysis without the variable *Age*, the variable *Wordlist (DemTect)* was selected as predictor, although in only one out of 50 iterations. This immediate recall of a wordlist probes short-term verbal memory. As discussed earlier, measures of memory capacity have been recurrently linked to speech-in-noise performance (cf. Akeroyd, 2008). In this context, the DemTect has not been discussed in the literature so far. Even though the DemTect is a short screening test for dementia, the separate subtests address specific cognitive domains. In a published conference proceeding, Meis et al. (2013) reported significant correlations between *SRT* and the memory-related subtests of the *DemTect* (*Wordlist, Wordlist delayed recall)*, the *Verbal fluency* subtest, and the overall sum score in middle-aged and elderly hearing-impaired individuals in an aided condition. This association, however, failed to yield significance in the unaided condition. In our study, the selection of the variable *Wordlist (DemTect)* did not add to the overall variance accounted for by the cognitive measures compared to the model with *Age*.

Among the other auditory measures, the variable *Loudness recruitment for 4 kHz (loudness scaling)* emerged as frequently selected predictor (33 of 50). Recruitment describes a phenomenon where the rate of perceived loudness with increasing level grows more rapidly than normal (Steinberg and Gardner, 1937; Hellman and Meiselman, 1993). It is possible that listeners show the same pure-tone audiogram reflecting deficits at threshold, i.e., at the limits of the auditory range, but may differ in loudness functions that additionally assess deficits for supra-threshold sounds at moderate levels. In contrast to levels at thresholds, moderate levels may be more related to daily life situations (cf. Brand and Hohmann, 2001, 2002). In this study, *Loudness recruitment* was defined as the slope of the lower part of the fitted loudness function using an Adaptive Categorical Loudness Scaling procedure (Brand and Hohmann, 2002; Oetting et al., 2014). The present result indicates that more recruitment results in poorer speech-recognition performance and underpins previous findings reporting an effect of loudness recruitment on speech recognition in noise (Villchur, 1974; Dreschler and Plomp, 1985; Moore and Glasberg, 1993). Likewise, a recent study by Van Esch and Dreschler (2015) identified *Loudness recruitment* as significant predictor for speech in fluctuating noise, yet accounting for only 3% additional variance following other auditory measures. In their study, this relationship did not persist for stationary noise. Our study contradicts the previous findings as it found a significant relationship for stationary noise. Yet, correspondingly, the additional predictive value of the two measures of the loudness scaling (*Loudness recruitment for 4 kHz* and *Hearing threshold level for 4 kHz*) in this study was marginal (<1%).

Moreover, several variables of the self-reports were recurrently selected as predictors, namely *Familial hearing loss* (48 of 50), *Sudden hearing loss* (30 of 50), *Subjective hearing problems in quiet* (24 of 50), *Subjective hearing problems in noise* (20 of 50), *Ear noise* (3 of 50), as well as *Hearing loss detected* (2 of 50) (see Supplementary Table S1 for explanations). While, with 6.6%, the relative contribution of these self-reported measures to the overall explained variance was the second highest after *PTA*, this might be attributed to the larger number of variables included in this domain relative to the other domains added in each step. Nonetheless, the present results suggest that auditory factors beyond pure-tone hearing thresholds, such as the occurrence of hearing loss in the family, the presence of ear noise (tinnitus) and the occurrence of a sudden hearing loss in the past, are related to inter-individual differences in speech-recognition performance. Furthermore, self-reported hearing problems seem relevant. Overall, findings on the relationship between subjective self-reports and the performance in “objective” speech-recognition tests vary across studies, ranging from no correlation to consistent correlations. Various factors have been investigated and discussed as possible mediators for hearing-impaired individuals, such as the use of hearing aids, the methods applied to obtain the signal-to-noise ratio, cognitive factors, and the equivalence of the specific listening situation addressed by both measures (cf. Heinrich et al., 2015, 2016; Heinrich and Knight, 2016). For older normal-hearings individuals, discrepancies between measured and self-assessed hearing difficulties have been discussed in the light of factors such as the experience of serious health issues, the amount of engegament in communication situations, and the relative evaluation of the normal-hearing elderly to their hearing-impaired age peers (Füllgrabe et al., 2015). Our results suggest that subjectively reported hearing problems, assessed with two short questions (*Subjective hearing problems in quiet* and *noise)*, contribute to variability in speech-in-noise performance, and might be of predictive value in addition to objective measures of hearing sensitivity as assessed by pure-tone audiometry.

These findings are also of interest in the light of the review by Knudsen et al. (2010) concluding that subjectively perceived hearing problems equally affected help seeking, hearing aid uptake, hearing aid use, and satisfaction with a hearing aid. In contrast, hearing threshold levels were related to help seeking and hearing aid uptake, but neither to the use of nor to the satisfaction with hearing aids. Furthermore, Moore et al. (2014) analyzed about half a million participants from the UK Biobank and found that hearing difficulties were more prevalent when assessed with self-reports than with objective measures (SRT), in the sense that even individuals with normal SRTs reported hearing difficulties. Taken together, these findings prompt that current objective speech reception tests might not be able to fully capture the difficulties subjectively experienced in everyday life situations, and that self-reports, especially self-reported subjective hearing problems, might add to the understanding of differences in speech-in-noise performance.

In addition to the aforementioned variables, the health-related measure *Physical sum score (SF-12)* (1 of 50) was selected, reflecting the self-reported physical health status of the individuals. It should be pointed out that the measure was the least frequently selected variable, and that its additional predictive value was marginal (<0.5%). This seems to relate to prior findings showing that speech perception measures correlate less with generic health questionnaires than with hearing-specific questionnaires (Chisolm et al., 2007; Heinrich et al., 2015).

Moreover, the *Grouping variable (Bisgaard)* was repeatedly selected as relevant predictor (27 of 50) suggesting that inter-individual differences in speech performance are linked to different audiogram profiles. Bisgaard et al. (2010) defined 10 standard audiograms covering the range of audiograms in clinical practice. The audiograms are proposed for the characterization of hearing aids that are programmed based on the actual user settings to minimize outcome variability. In contrast to the *PTA* typically using the average across the four frequencies 0.5, 1, 2, and 4 kHz, these audiograms consider the profile across 10 frequencies ranging from 0.25 to 8 kHz. These profiles allow a categorization of hearing loss based on the degree (*very mild* to *severe*) and additionally, based on the slope of the profile (*flat/moderately sloping, steep sloping*) (cf. Bisgaard et al., 2010). The present findings indicate that the audiogram profile relates to differences in speech-in-noise performance with an additional relative contribution of 2.6% when entered lastly after the other measures. This motivated us to conduct a complementary analysis, where we investigated these groups in more detail by categorizing the participants with respect to both hearing aid use and hearing loss profiles.

## Complementary Analysis

In this complementary analysis, we were interested in the question whether hearing-aid users (HAU) differ from those with the same hearing loss profile who have not been provided with a hearing aid yet, the so-called NU, regarding the test measures and the regressor variables for speech recognition in noise.

### Materials and Methods

As the aim of the complementary analysis was to compare HAU to NU with the same hearing loss profile, we first categorized the 438 participants from the main analysis based on their current status of hearing aid use. Current hearing aid supply (i.e., ownership) and use (i.e., experience) was assessed in the questionnaire and verified in the anamnesis. We aimed at categorizing the individuals regarding their status of hearing aid use and experience, and correspondingly, categorized individuals who own a hearing aid but never use it as NU. Thus, we applied the variable *Hearing aid use (current status)* as categorization criterion for HAU and NU. 93% of the HAU indicated that they used their hearing aids every day during the last 14 days, with an average daily wearing period of 6 h, supporting that most of HAU indeed use their hearing aid(s) and are experienced. All HAU were included, irrespective of one- or two-sided hearing aids, the duration of hearing aid supply, and the daily wearing period. To characterize HAU and NU, the *Sex* and *Age*, as well as the *PTA* and *50*%*-SRT* distributions are shown in **Table 3**.

**Table 3:** Characteristics of hearing aid users (HAU) and non-users (NU).

	HAU *n* = 223	NU *n* = 215	Total *n* = 438
Males, n (%)	156 (70.0)	116 (54.0)	272 (62.1)
Females, n (%)	67 (30.0)	99 (46.0)	166 (37.9)
Age in years, mean (SD)	71.9 (6.2)	70.2 (5.2)	71.1 (5.8)
PTA in dB HL, mean (SD)	43.5 (12.3)	23.8 (9.6)	33.8 (14.8)
50%-SRT in dB SNR, mean (SD)	-0.9 (3.0)	-1.2 (3.1)	-1.1 (3.1)

*Shown are frequencies of the gender distribution of HAU and NU. Furthermore, the mean value and the standard deviation of the *Age, PTA* and *50*%*-SRT* distributions among HAU and NU are presented. SD, standard deviation.*

**FIGURE 4:**
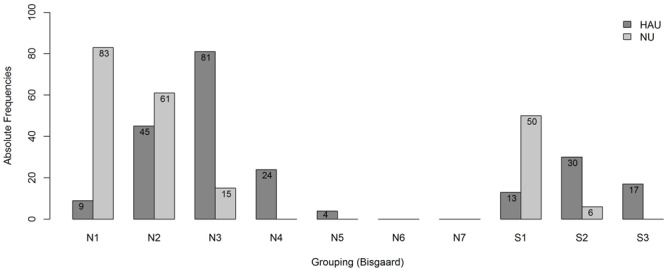
**Frequency distribution of the subgroups.** Shown are absolute frequencies of the groups N1,…, N7 and S1, S2, S3 among HAU and NU of in total 438 participants.

To account for differences in the degree of hearing impairment between the two groups and motivated by the results of the main analysis, we further classified the individuals with respect to their audiogram profiles according to Bisgaard et al. (2010), cf. **Figure 2**, main analysis. This two-step categorization allowed for a groupwise comparison between HAU and NU showing the same type of hearing impairment in terms of their audiogram profile. This resulted in 13 subsets (10 audiogram categories for each of the two groups, HAU and NU, of which seven were empty), see **Figure 4**. Due to the small sample sizes in some subgroups and unbalanced group sizes between HAU and NU, we only considered the individuals in subgroup N2, i.e., those with a *mild* and *flat/moderately sloping* hearing loss, for this analysis. Thus, we compared HAU-N2 (*n* = 45) to NU-N2 (*n* = 61) regarding the test measures and the regressor variables for speech recognition in noise.

### Analysis

#### Groupwise Comparison

In a first step, we investigated potential group differences regarding the test measures between HAU and NU with the audiogram profile N2. To that end, pairwise comparisons were conducted using independent samples Mann–Whitney-*U*-Tests on the test measure variables. As this involved multiple pairwise comparisons, the Bonferroni correction was used to maintain the Type I error rate at 0.05 and the adjusted *p*-values are reported.

#### Groupwise Regression Analyses

Secondly, the stepwise linear regression algorithm was applied for the two subgroups in order to characterize HAU and NU comprising the same hearing loss profile in terms of explaining measures for speech recognition. As the distribution of the *50*%*-SRT* was more symmetric within the two groups, we did not transform the outcome variable for the regression analyses at group level. Furthermore, none of the potential regressor variables was transformed to avoid different transformations for one regressor across the groups, thus, yielding comparable results. Finally, in contrast to the main analysis, the cross-validation was omitted in the group analyses as the sample size of the analyzed groups was considered too small for performing a *k*-fold cross-validation. According to Kuhn and Johnson (2013), *k*-fold cross-validation generally has potential issues with high variance and bias that become negligible only for large training sets.

As potential regressor variables, the same variables as described in the main analysis were taken into account except for the two variables that were used for categorization (*Grouping variable (Bisgaard), Hearing aid use (current status)*), and the variable *Duration of hearing aid supply* as this variable was only meaningful for HAU.

### Results

#### Groupwise Comparison

**FIGURE 5:**
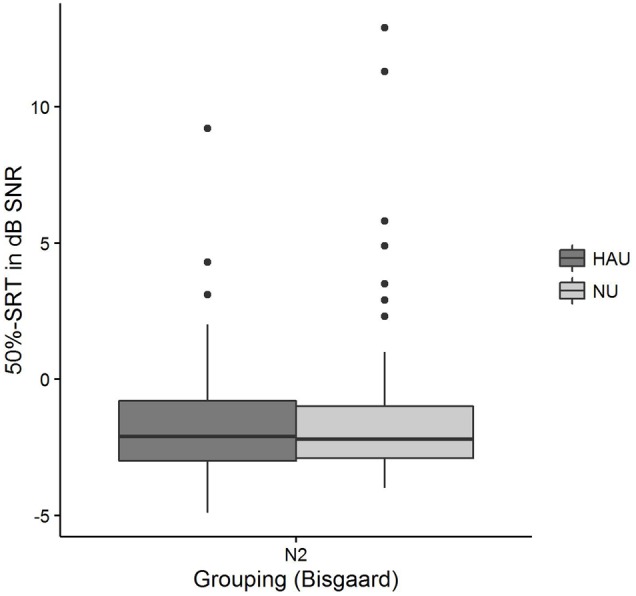
***50*%*-SRT* distributions among the two subgroups.** Figure shows boxplots for the test measure *50*%*-SRT*.

**FIGURE 6:**
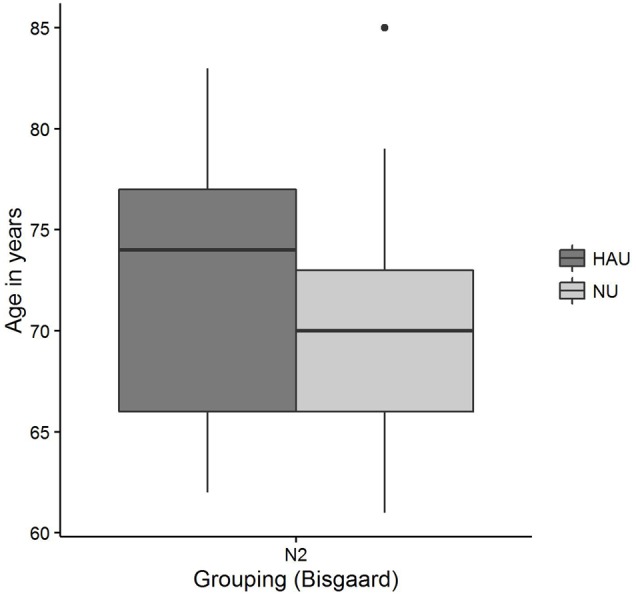
***Age* distributions among the two subgroups.** Figure shows boxplots for the test measure *Age*.

**FIGURE 7:**
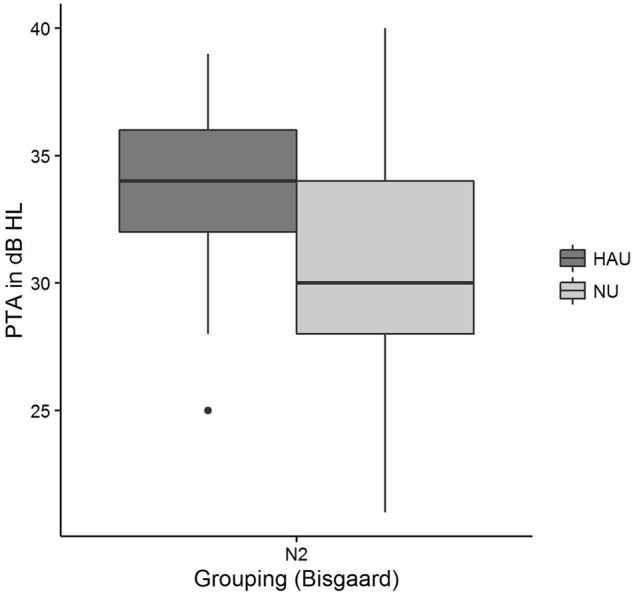
***Pure-tone average* distributions among the two subgroups.** Figure shows boxplots for the test measure *PTA*.

The distributions of *50*%*-SRT, Age*, and *PTA* are visualized in **Figures 5**–**7**. With respect to the outcome variable *50*%-*SRT*, no significant differences were found between HAU-N2 and NU-N2 regarding the three subgroups, respectively (see **Figure 5**). Thus, the unaided speech-recognition performance of HAUs was on par with the performance of NU with the same mild hearing loss. Likewise, groups did not differ significantly in the variable *Age*, as shown in **Figure 6**.

Pairwise comparisons revealed significant group differences for the auditory variables *PTA* (*p*_adj_ < 0.001) (see **Figure 7**), *Hearing threshold level for 4 kHz (loudness scaling)* (*p*_adj_ < 0.001), and *Hearing threshold level for 1.5 kHz (loudness scaling)* (*p*_adj_ < 0.001). More precisely, HAU-N2 showed higher values than NU-N2 in all these variables. Furthermore, HAU-N2 and NU-N2 differed significantly in the self-reported measure *Subjective hearing problems in quiet* (*p*_adj_ < 0.001), such that HAU-N2 reported more subjective hearing problems without hearing aids than NU-N2. The other auditory, cognitive and self-reported measures did not reveal significant differences.

#### Groupwise Regression Analyses

The results of the two linear regression models are shown in **Table 4**. For HAU-N2, 35% of the variance in the *50*%*-SRT* was explained by the model. *Verbal intelligence* emerged as the most important regressor as the stepBIC algorithm selected this variable first (*p* < 0.001) and explained 15% variance. Secondly, the health measure *Multimorbidity sum score* was selected (*p* < 0.01) explaining 14%, and lastly, the subjectively reported measure *Hearing loss detected* (*p* < 0.05) with 6% accounted variance. In the group NU-N2, 58% of the variability was accounted for by six regressors. *Age* emerged as first predictor (*p* < 0.001) explaining 27% variance. Among the remaining regressors were two subjectively reported auditory measures (*Sudden hearing loss, p* < 0.001*; Hearing loss detected, p* < 0.01), two auditory ones [*PTA, p* < 0.05; *Uncomfortable loudness level for 4 kHz (loudness scaling), p* < 0.05] and the cognitive measure *Verbal intelligence* (*p* < 0.01).

**Table 4:** Regression models for the groups HAU-N2 and NU-N2.

Group	Regressors		β	*SE*	*p*	Δ*R*^2^
HAU-N2	Intercept		2.29	1.80	0.21	-
	*Verbal intelligence*		-0.18	0.05	<0.001^∗∗∗^	0.15
	*Multimorbidity sum score*		-0.15	0.05	0.004^∗∗^	0.14
	*Hearing loss detected*	Yes	3.04	1.38	0.03^∗^	0.06
						**Total *R*^2^ = 0.35**

NU-N2	Intercept		-10.40	1.55	<0.001^∗∗∗^	-
	*Age*		0.08	0.02	<0.001^∗∗∗^	0.27
	*Sudden hearing loss*	Yes	0.83	0.19	<0.001^∗∗∗^	0.09
	*Verbal intelligence*		-0.06	0.02	0.001^∗∗^	0.08
	*Hearing loss detected*	Yes	0.83	0.27	0.003^∗∗^	0.06
	*Uncomfortable loudness level for 4 kHz (loudness scaling)*		0.02	0.01	0.01^∗^	0.04
	*PTA*		0.05	0.02	0.02^∗^	0.04
						**Total *R*^2^ = 0.58**

*Shown are the regression results of the group analysis comparing HAU with NU having the same audiogram profile N2 (*mild* and *flat/moderately sloping* hearing loss).*

*β = estimated regression coefficient.*

*SE = estimated standard deviation (standard error) of β.*

*p = *p*-value for the two-tailed *t*-test of the null hypothesis that a coefficient equals zero. Significant codes: ^∗∗∗^*p* < 0.001, ^∗∗^*p* < 0.01, ^∗^*p* < 0.05.*

*R^*2*^ = adjusted coefficient of determination.*

*Δ*R*^*2*^ = change in the amount of *R*^*2*^ by adding the respective regressor in the order of variable selection by stepBIC reflecting the proportion of explained variance accounted for as the respective regressor is added to the model.*

### Discussion

In this complementary analysis, we compared HAU and NU with the same mild hearing loss profile regarding group differences in the test measures and regarding the regressor variables for speech recognition in noise. To allow a comparison between HAU and NU, we first grouped the 438 individuals of the main analysis based on their current status of hearing aid use into HAU and NU. Secondly, to avoid the confound of stronger hearing impairment in one group and motivated by the results of the main analysis, we matched those groups based on their audiogram profile and only compared HAU and NU with the same audiogram profile (N2).

#### Groupwise Comparison

First, we probed whether there are any significant differences with respect to the test measures between HAU and NU having the same hearing loss profile.

Group comparisons for individuals with a *mild, flat/moderately* sloping hearing loss (N2) revealed significant differences for the *PTA, Hearing threshold level for 1.5* and *4 kHz (loudness scaling)*, as well as *Subjective hearing problems in quiet*. Hence, HAUs with a mild hearing loss differed from NU mainly with respect to auditory-related measures, a result that is particularly interesting given the fact that the groups were matched based on the audiogram profile, thus showing the same degree and pattern of hearing loss. The results indicate that although having similar *SRTs*, these groups still differ in their hearing thresholds as derived from the categorical loudness scaling. There is evidence for adaptive functional plasticity in the top-down regulation and perception of loudness in HAUs (Philibert et al., 2005; Munro et al., 2007), indicating that stimuli are perceived less loud (Munro and Merrett, 2013) and that levels of uncomfortable loudness levels are elevated (Munro and Trotter, 2006) following hearing aid experience. In our analysis, there was a trend for group differences for the variables *Loudness recruitment for 1* and *4 kHz (loudness scaling)* (*p* < 0.05), as well as for the variables *Medium loudness level for 1* and *4 kHz (loudness scaling)* (*p* < 0.05), such that HAU showed a trend for higher values. When applying Bonferroni correction, however, these differences were no longer significant. Likewise, no significant differences were found for the *Uncomfortable loudness level*. Nevertheless, elevated hearing thresholds in HAU might point toward plasticity in the auditory system.

Despite the same audiogram profile and similar *50*%*-SRT*s, HAU with a mild hearing loss also showed higher self-reported *Subjective hearing problems in quiet* (without hearing aids). While the results of the main analysis indicated that subjectively reported hearing difficulties are linked to speech-in-noise performance, this relationship could be mediated by the underlying degree of hearing loss. The present group analysis prompts that groups matched regarding their degree of hearing loss still display differences in these subjectively reported measures. This relates to previous studies showing that individual perception of hearing problems influenced, among others, help seeking as well as hearing aid uptake and use (van den Brink et al., 1996; Duijvestijn et al., 2003; Humes, 2003). Accordingly, individuals subjectively perceiving more hearing problems are more likely to seek help and acquire hearing aids, concluding that self-reported hearing problems might be more relevant for auditory rehabilitation than objective hearing sensitivity measures (for review see Knudsen et al., 2010). Whether in our study the HAU already experienced more hearing difficulties prior to their hearing aid acquisition or whether they were more aware of their own hearing problems without the hearing aid at the moment of being questioned, is not clear, however.

Nevertheless, individuals with lower levels of subjective hearing loss might already benefit from a hearing aid as studies have shown that many people who could benefit from hearing aids are not supplied (Smeeth et al., 2002; Smits et al., 2006), especially those with a mild hearing impairment (Lin et al., 2011; Chien and Lin, 2012). This is of importance since an uncorrected hearing loss has been linked to lower quality of life, reduced social activity, isolation, and increased symptoms of depression (for review see Arlinger, 2003). In comparison, our group comparisons did not show differences regarding the *Mental sum score* of the *SF-12*, comprising subscales of vitality, social functioning, role emotional, and mental health, thus not giving evidence that hearing aid use is associated with higher MCSs.

Likewise, our group comparisons did not reveal significant differences in any of the cognitive measures. Prior studies have shown that hearing loss is independently associated with cognitive decline (Lin et al., 2013), and several studies have investigated the link between hearing aid use and cognitive performance. Kalluri and Humes (2012) reviewed the available data on the relationship between cognitive performance and hearing technology and found supporting evidence for an effect of hearing aids on immediate cognitive functions. Regarding the long-term effects of hearing aid use, however, data is limited and equivocal, thus giving little evidence for long-term protective effects against cognitive decline, mainly due to missing appropriate longitudinal studies or small sample sizes. This has recently been complemented by a prospective 25-year longitudinal study suggesting that hearing aid use does attenuate cognitive decline in elderly people in the sense that hearing-impaired individuals wearing hearing aids showed similar rates of cognitive decline as normal-hearing subjects in contrast to those without hearing aids (Amieva et al., 2015). Their results further suggest that hearing loss is not directly related to cognitive decline but, rather, mediated via factors of social isolation and depression. However, as this was an observational study, other factors than hearing-aid use could explain these results.

As the present study is only a cross-sectional snapshot, we cannot draw conclusions on whether hearing aid use is independently associated with cognitive decline or not. The results merely indicate that the investigated groups of HAU and NU with a mild hearing loss did not differ *per se* with respect to their cognitive abilities when being matched with respect to age and pattern of hearing loss. In the light of possible mediators of social isolation and depression discussed earlier, the missing differences regarding the *Mental sum score* (*SF-12)* might relate to missing differences in cognitive abilities. Another possibility that should be outlined is the focus of this analysis on individuals with a mild hearing loss only to be able to compare HAU to NU with a matched hearing loss profile. It is possible that more severe hearing impairments might yield a different picture.

Lastly, it is to point out that the HAU did not differ from the NU in terms of their *Socio-economic status sum score.* This is compliant to prior findings indicating hearing aid acquisition/uptake is mostly unrelated to the socio-economic position (Knudsen et al., 2010; Benova et al., 2015).

#### Groupwise Regression Analyses

Within the aim of the complementary analysis, we were further interested whether HAU and NU with the same hearing loss profile differ regarding the regressor variables unaided speech recognition. First, the overall picture shows that *Verbal intelligence* and *Hearing loss detected* emerged as relevant regressors in both groups while the other regressor variables differed. This indicates that the relevant measures might constitute a function of both hearing aid use and audiogram profile. Overall, the selected regressors explained a total of 35% in *SRT* for HAU-N2 and 58% for NU-N2.

In conformity with the results of the main analysis, the cognitive measure *Verbal intelligence* emerged as highly significant variable in both groups such that higher scores in *Verbal intelligence* were associated with better speech-in-noise performance. Being selected as first (HAU-N2) and tertiary (NU-N2) regressor, *Verbal intelligence* showed a predictive value of 15 and 8%, respectively. The subjectively reported measure *Hearing loss detected* was likewise among the selected regressors in both groups, contributing in each with 6% to the overall variance. This variable might be thought of as an additional indicator of the severity of (subjective) hearing loss.

In the HAU-N2 group, the health measure *Multimorbidity sum score* contributed to the *SRT* prediction in the sense that individual with more self-reported medical conditions showed lower speech-in-noise performance. In contrast to the main analysis, the health measure had a substantial predictive value for HAU. With 14% it explained almost as much as the cognitive measure.

In the NU-N2 group, on the other hand, *Age* constituted the most important regressor explaining the most variance (27%). While this is compliant to the results by Houtgast and Festen (2008) showing correlations between *SRT* and *Age* after controlling for the degree of hearing loss in terms of *PTA*, it cannot explain why *Age* is only selected in the NU-N2 but not in the HAU-N2 group. Among the remaining selected regressors in the NU-N2 group was another subjectively reported measure (*Sudden hearing loss*) accounting for 9%, and two auditory measures [*PTA, Uncomfortable loudness level for 4 kHz (loudness scaling)*], together explaining 8%. While it might appear surprising that the *PTA* entered as significant regressor only in the NU group, it is to point out that the groups were controlled for degree of hearing loss such that the individuals in one group displayed the same degree of hearing loss according to the aforementioned Bisgaard categorization. Apparently, *PTA* emerged as significant regressor for the NU-N2 group as it showed a higher variability in *PTA* with a number of individuals having *PTA* values below 26 despite this classification (see **Figure 7**). Thus, a classification based on the *PTA* according to WHO guidelines might label those individuals as normal-hearing (WHO, 1991), whereas a classification considering the more comprehensive audiogram profile might lead to the classification of a mild hearing loss. This suggests that in contrast to using the proposed audiogram profiles, the *PTA* might not fully capture rather mild degrees of hearing loss, which should be considered in the context of diagnosis and hearing aid prescription as this group might already benefit from hearing aids.

The variable *Uncomfortable loudness level for 4 kHz* measured with the loudness scaling additionally came up as significant regressor for NU-N2 in the sense that higher thresholds of experienced loudness discomfort were associated with a higher *50*%*-SRT* scores, thus lower speech intelligibility. While standard measures of pure-tone audiometry such as *PTA* focus on hearing thresholds, *Uncomfortable Loudness Level* constitutes a supra-threshold measure of loudness perception. The present findings suggest that measures of loudness discomfort might add to the variability in *50*%*-SRT* for hearing impaired individuals who are not yet provided with hearing aids. This might also be relevant in the light of previous studies that have used supra-threshold loudness perception measured by the Categorical Loudness Scaling to fit hearing aids (Kiessling et al., 1996; Valente and Van Vliet, 1997; Herzke and Hohmann, 2005). In contrast to the main analysis, recruitment was not selected as a relevant regressor, although this supra-threshold measure might be related even more to communication (cf. Brand and Hohmann, 2001, 2002).

When contrasting the amount of overall explained variance to the main analysis, it becomes apparent that particularly in the HAU group, there is a substantial amount of variability unaccounted for. This points toward relevant measures that have been missed and which are brought up in the overall discussion.

## Overall Discussion

Summing up, the full-data regression in the main analysis confirmed previous findings showing that *PTA* and *Age* are among the primary predictors for unaided speech recognition in noise, and generalized this result to a large sample of hearing-impaired individuals with various patterns of hearing loss, with and without hearing aid experience.

Furthermore, cognitive abilities were equally selected as primary predictor, although explaining marginal additional variance. Unlike previous studies, we found a consistent association between speech-in-noise performance and *Verbal intelligence*, an indicator of crystallized intellectual abilities loading highly on a general intelligence factor. In contrast, this study did not reveal an association with memory-related measures. When excluding *Age* as a potential correlate from the analysis, a measure of short-term verbal memory is selected as last predictor, however, together with *Verbal intelligence* only contributing 1.51% to the overall explained variance. One possible explanation for this discrepancy to previous studies, which repeatedly found significant correlations between speech intelligibility and working memory, might be the use of a different test. While this link has been most consistently found when working memory was assessed by means of the complex Reading Span test (Akeroyd, 2008), we used the simpler *Digit reverse* subtest tapping into working memory and the *Wordlist* subtest tapping into short-term verbal memory, which were part of our dementia screening test (DemTect). Moreover, the fact that no single cognitive component always showed a significant association to speech in noise across prior studies has been discussed with respect to the variability in complexity of the cognitive tasks as well as to the variations regarding the speech-in-noise task and specific listening situations (Heinrich et al., 2015; Heinrich and Knight, 2016). Another rationale behind the comparably low predictive value of *Verbal intelligence* and the missing effects of memory-related measures might lie in the *unaided* measurements. As *aided* measurements have revealed stronger associations between memory and SRT than *unaided* ones (Meis et al., 2013), it is possible that the predictive value of cognitive abilities becomes more evident when the peripheral hearing loss is compensated for.

The present results further suggest that auditory measures beyond pure-tone audiometry such as loudness recruitment and auditory-related self-report measures have an additional predictive value for unaided speech-in-noise prediction. Moreover, classifying the hearing-impaired individuals with respect to their specific audiogram profiles seems reasonable. The applied categorization takes into account different slopes of the audiogram such as the *steep sloping* hearing loss which might be concealed when using the *PTA* for classification.

Despite the wide range of included measures, the overall prediction left 38% variance unexplained suggesting that further relevant measures might have been missed. This can be attributed to the fact that the test battery was originally designed for a characterization of individuals from a cohort and not primarily for unaided speech-in-noise prediction. Other factors that have been linked to speech recognition before and were not included in the battery comprise, among others, measures of spectral and temporal processing (Noordhoek et al., 2001; George et al., 2006, 2007; Füllgrabe, 2013; Füllgrabe et al., 2015), and in the cognitive domain, inhibition (Sommers and Danielson, 1999; Janse, 2012), linguistic abilities (Avivi-Reich et al., 2014), as well as attention (Mattys and Palmer, 2015; Heinrich et al., 2016). Besides cognition, recent evidence points toward a significant contribution of central auditory processing, measured by auditory brainstem responses, to understand speech in noise (Anderson et al., 2013a,b).

When contrasting HAUs with NU in the context of the complementary analysis, the groupwise comparisons regarding the regressor variables revealed significant differences between HAU and NU with a mild hearing loss with respect to auditory measures and self-reports. These differences should be taken into account in the context of hearing aid prescription and hearing aid fittings. Hearing-impaired individuals who are not yet supplied with a hearing aid subjectively perceive less hearing problems, nevertheless they might already benefit from a hearing aid. This suggests that the awareness of one’s own hearing problems is of particular relevance for further help seeking and hearing aid acquisition. Thus, measures of subjective hearing problems should take a more prominent role in the future.

Some limitations should be underlined such as the observational character of these analyses. Initially, data were collected in order to characterize normal-hearing and hearing-impaired individuals, which explains the selection of the corresponding auditory and non-auditory measures. Besides the facts that secondary data were used for the analyses and that this is not a prospective study, HAU were largely overrepresented in the sample and it cannot be ruled out that the sample differed from the population in a systematic way in other aspects as well, as self-motivated hearing-aid uptake might be associated with individual characteristics that also affect speech-in-noise recognition (cf. Knudsen et al., 2010). This should be taken into account when interpreting the results. The same holds for the fact that the hearing loss comprised forms of both symmetric and asymmetric hearing loss. Furthermore, as the *50*%*-SRT* measurement was unaided, the results may be different for aided measurements. Furthermore, groupwise analyses only focused on groups with a mild hearing impairment and can thus not be generalized to more severe forms.

The strength of this work, on the other hand, lies in the large overall sample size which allows for a generalizability of the results regarding the overall regression analysis of the main analysis. Moreover, we included a wide range of auditory, cognitive and self-report measures simultaneously, thus being able to specifically identify relevant measures in terms of additional predictive value. At the same time, these measures are suitable for the clinical setting with a total measurement time of approximately 1 h. Furthermore, the present classification based on audiogram profiles gives a more comprehensive picture compared to the commonly used PTA by considering a wider range of frequencies and different slopes.

## Author Contributions

AG and MT conceptualized the research question, analyzed the data, interpreted the results and wrote the manuscript. Data acquisition was conducted by the Hörzentrum Oldenburg GmbH under the responsibility of KW. CT and HC supervised the work. CT, KW, MM, and HC critically reviewed and significantly contributed to the manuscript. All authors approved the final version of the manuscript for publication. All authors agree to be accountable for all aspects of the work and in ensuring that questions related to the accuracy or integrity of any part of the work are appropriately investigated and resolved.

## Conflict of Interest Statement

The authors declare that the research was conducted in the absence of any commercial or financial relationships that could be construed as a potential conflict of interest.

## Footnotes

1 All variables entering the analyses are described in the Supplementary Table S1 and are written in italic letters.
