# Supplementary material for: Auditory and Non-Auditory Contributions for Unaided Speech Recognition in Noise as a Function of Hearing Aid Use
Source: Front Psychol. 2017 Feb 21;8:219. doi: 10.3389/fpsyg.2017.00219 (PMC5318449; doi:10.3389/fpsyg.2017.00219)
Supplement: Supplementary file 1 [file Table_1.docx]

**Supplementary Table S1. List of all auditory and non-auditory measures of the test battery taken into account for the statistical analyses. (A) Continuous variables summarized descriptively.** Column “No” = Variable number; Column “Type” = Type of variable, either outcome or predictor variable; Column “Subject Area” = Subject area of variable: Auditory, Demographic, Self-reports, Cognitive, Health, Economic-technical or Grouping; Column “Variable” = Variable name; Column “Description” = More detailed variable information; Column “Transformation” = Transformation by taking the logarithm, square root or reciprocal as indicated; Column “Unit” = Units of the variables, also of the transformed ones; Columns “Mean” – “Max” = Summary statistics of the variables; Column “NA” = Number of missing values; Column “Cor” = Pearson correlation of (untransformed) outcome variable *50%-SRT* and respective predictor variable; Column “p-value” = p-value of Pearson’s correlation test (two-sided); **(B) Categorical variables with their frequency distributions**. Columns “No” – “Description”, identical as described in (A); Column “Frequencies (percentages)” = Frequency and percentage distributions of the variables; Column “NA”, identical as described in (A).

**(A) Continuous variables summarized descriptively**

| **No** | **Type** | **Subject Area** | **Variable** | **Description** | **Transformation** | **Unit** | **Mean** | **Sd** | **Min** | **Q1** | **Median** | **Q3** | **Max** | **NA** | **Cor** | **p-value** |
| --- | --- | --- | --- | --- | --- | --- | --- | --- | --- | --- | --- | --- | --- | --- | --- | --- |
| 1 | Outcome | Auditory | *50%-SRT* | Signal-to-noise ratio for 50% speech intelligibility (Goettingen sentence test) | logarithm | dB SNR  log(dB SNR) | -1.0    2.2 | 3.0    0.3 | -5.1    1.6 | -2.9  2.0 | -2.0  2.1 | 0    2.3 | 15.7    3.2 | 0  0 | -  - | -  - |
| 2 | Predictor | Auditory | *PTA* | Pure-tone average (better ear: 0.5,1,2,4 kHz; audiogram) |  | dB HL | 33.8 | 14.8 | 4.0 | 21.3 | 34.0 | 44.0 | 74.0 | 0 | 0.74 | <0.01 |
| 3 | Predictor | Auditory | *Uncomfortable loudness level for 0.5 kHz (audiogram)* |  |  | dB HL | 95.7 | 11.6 | 45.0 | 90.0 | 95.0 | 100.0 | 115.0 | 5 | 0.14 | <0.01 |
| 4 | Predictor | Auditory | *Uncomfortable loudness level for 1 kHz (audiogram)* |  |  | dB HL | 94.7 | 10.6 | 60.0 | 90.0 | 95.0 | 100.0 | 120.0 | 2 | 0.18 | <0.01 |
| 5 | Predictor | Auditory | *Uncomfortable loudness level for 2 kHz (audiogram)* |  |  | dB HL | 97.9 | 10.1 | 60.0 | 90.0 | 98.5 | 105.0 | 120.0 | 3 | 0.31 | <0.01 |
| 6 | Predictor | Auditory | *Uncomfortable loudness level for 4 kHz (audiogram)* |  |  | dB HL | 99.7 | 10.3 | 55.0 | 95.0 | 100.0 | 105.0 | 120.0 | 12 | 0.32 | <0.01 |
| 7 | Predictor | Auditory | *Hearing threshold level for 1.5 kHz (loudness scaling)* | Level at 2.5 CU for 1.5 kHz |  | dB HL | 31.0 | 17.8 | -5.0 | 17.5 | 31.1 | 44.8 | 82.3 | 1 | 0.62 | <0.01 |
| 8 | Predictor | Auditory | *Medium loudness level for 1.5 kHz (loudness scaling)* | Level at 25 CU for 1.5 kHz | logarithm | dB HL  log(dB HL) | 82.4  4.4 | 7.9    0.1 | 51.0  3.9 | 77.8  4.4 | 82.4  4.4 | 86.6  4.5 | 140.4  4.9 | 1  1 | 0.34  0.33 | <0.01  <0.01 |
| 9 | Predictor | Auditory | *Uncomfortable loudness level for 1.5 kHz (loudness scaling)* | Level at 50 CU for 1.5 kHz | square root | dB HL  sqrt(dB HL) | 104.0  10.2 | 8.0    0.4 | 81.1  9.0 | 98.8  9.9 | 103.2  10.2 | 108.0  10.4 | 161.1  12.7 | 1  1 | 0.16  0.16 | <0.01  <0.01 |
| 10 | Predictor | Auditory | *Hearing threshold level for 4 kHz (loudness scaling)* | Level at 2.5 CU for 4 kHz |  | dB HL | 50.0 | 20.3 | -5.0 | 35.8 | 53.0 | 64.3 | 100.0 | 0 | 0.53 | <0.01 |
| 11 | Predictor | Auditory | *Medium loudness level for 4 kHz (loudness scaling)* | Level at 25 CU for 4 kHz |  | dB HL | 88.9 | 10.3 | 12.4 | 82.9 | 88.4 | 94.3 | 130.7 | 0 | 0.35 | <0.01 |
| 12 | Predictor | Auditory | *Uncomfortable loudness level for 4 kHz (loudness scaling)* | Level at 50 CU for 4 kHz | square root | dB HL  sqrt(dB HL) | 107.3  10.3 | 10.1  0.5 | 74.4  8.6 | 100.5  10.0 | 106.2  10.3 | 112.9  10.6 | 156.8  12.5 | 0  0 | 0.29  0.30 | <0.01  <0.01 |
| 13 | Predictor | Auditory | *Loudness recruitment for 1.5 kHz (loudness scaling)* | Slope of the lower part of the fitted loudness function for 1.5 kHz | reciprocal | CU/dB  dB/CU | 0.5  2.4 | 0.2    0.8 | 0.2  0.5 | 0.3  1.8 | 0.4  2.4 | 0.6  3.0 | 2.2  5.0 | 1    1 | 0.47  -0.51 | <0.01  <0.01 |
| 14 | Predictor | Auditory | *Loudness recruitment for 4 kHz (loudness scaling)* | Slope of the lower part of the fitted loudness function for 4 kHz | logarithm | CU/dB  log(CU/dB) | 0.7  -0.5 | 0.5  0.5 | 0.2  -1.6 | 0.4  -0.8 | 0.6  -0.5 | 0.8  -0.3 | 3.8  1.3 | 0    0 | 0.38  0.47 | <0.01  <0.01 |
| 15 | Predictor | Demographic | *Age* | Age at the time of the face-to-face interview (only year and month of birth were given) |  | years | 71.1 | 5.8 | 60.0 | 66.0 | 71.0 | 75.0 | 85.0 | 0 | 0.22 | <0.01 |
| 16 | Predictor | Cognitive | *Wordlist (DemTect)* | DemTect subtest wordlist |  | points | 13.0 | 2.6 | 5.0 | 11.0 | 13.0 | 15.0 | 20.0 | 0 | -0.22 | <0.01 |
| 17 | Predictor | Cognitive | *Verbal fluency (DemTect)* | DemTect subtest supermarket task |  | points | 23.3 | 5.0 | 2.0 | 20.0 | 23.0 | 28.0 | 30.0 | 0 | -0.19 | <0.01 |
| 18 | Predictor | Cognitive | *Wordlist delayed recall (DemTect)* | DemTect subtest wordlist delayed recall |  | points | 4.9 | 2.2 | 0.0 | 4.0 | 5.0 | 6.0 | 10.0 | 0 | -0.15 | <0.01 |
| 19 | Predictor | Cognitive | *Verbal intelligence* | Raw score of Wortschatztest |  | points | 31.4 | 5.1 | 8.0 | 29.0 | 32.0 | 35.0 | 41.0 | 0 | -0.25 | <0.01 |
| 20 | Predictor | Health | *Physical sum score (SF-12)* |  |  | points | 46.4 | 9.2 | 18.7 | 40.8 | 49.1 | 53.8 | 62.1 | 8 | -0.06 | 0.23 |
| 21 | Predictor | Health | *Mental sum score (SF-12)* |  |  | points | 52.5 | 8.5 | 21.9 | 50.4 | 55.2 | 57.9 | 67.6 | 8 | -0.06 | 0.18 |
| 22 | Predictor | Health | *Multimorbidity sum score* |  | logarithm | points  log(points) | 6.0  1.6 | 3.7    0.6 | 2.0  0.7 | 4.0  1.4 | 6.0  1.8 | 6.0  1.8 | 28.0  3.3 | 80  80 | -0.05  -0.05 | 0.33  0.26 |
| 23 | Predictor | Economic-technical | *Socio-economic status sum score* |  |  | points | 13.0 | 3.9 | 5.0 | 10.0 | 12.0 | 17.0 | 21.0 | 3 | -0.13 | <0.01 |

**(B) Categorical variables with their frequency distributions**

| **No** | **Type** | **Category** | **Variable** | **Description** | **Frequencies (percentages)**  **n = 438** | **NA** |
| --- | --- | --- | --- | --- | --- | --- |
| 24 | Predictor | Demographic | *Sex*  Male  Female | Gender | 272 (62.1%)  166 (37.9%) | 0 |
| 25 | Predictor | Self-reports | *Middle ear infection often on the left side*  Yes  No  Unknown | Q: Have you often had a middle ear infection on the left side? | 46 (10.5%)  384 (87.7%)  8 (1.8%) | 2 |
| 26 | Predictor | Self-reports | *Middle ear infection often on the right side*  Yes  No  Unknown | Q: Have you often had a middle ear infection on the right side? | 47 (10.7%)  383 (87.4%)  8 (1.8%) | 2 |
| 27 | Predictor | Self-reports | *Progressive hearing loss*  Yes  No  Unknown | Q: Is your hearing loss progressive? | 161 (36.8%)  243 (55.5%)  34 (7.8%) | 4 |
| 28 | Predictor | Self-reports | *Fluctuating hearing loss*  Yes  No  Unknown | Q: Is your hearing ability fluctuating? | 38 (8.7%)  387 (88.4%)  13 (3.0%) | 4 |
| 29 | Predictor | Self-reports | *Hearing aid use (current status)*  Yes  No | Q: Do you currently use a hearing aid? | 223 (50.9%)  215 (49.1%) | 1 |
| 30 | Predictor | Self-reports | *Duration of hearing aid supply*  0-9 years  10-19 years  20-29 years  30-39 years  40-49 years  >= 50 years  Not asked | Q: How long have you been using hearing aids? | 147 (33.6%)  60 (13.7%)  13 (3.0%)  4 (0.9%)  2 (0.5%)  1 (0.2%)  211 (48.2%) | 4 |
| 31 | Predictor | Self-reports | *Familial hearing loss*  No  Yes  Unknown | Q: Does hearing loss occur often in your family? | 262 (59.8%)  98 (22.4%)  78 (17.8%) | 8 |
| 32 | Predictor | Self-reports | *Native language German*  Yes  No |  | 435 (99.3%)  3 (0.7%) | 0 |
| 33 | Predictor | Self-reports | *Hearing loss detected*  No  Yes | Q: Have you, a doctor, relatives or friends determined that you have a hearing problem? | 72 (16.4%)  366 (83.6%) | 0 |
| 34 | Predictor | Self-reports | *Duration of hearing loss, left ear*  0-9 years  10-19 years  20-29 years  30-39 years  40-49 years  >= 50 years  Not asked | Q: If yes, how long has it been since the hearing problems were determined for the first time (left ear)? | 108 (24.7%)  211 (48.2%)  25 (5.7%)  11 (2.5%)  6 (1.4%)  5 (1.1%)  72 (16.4%) | 158 |
| 35 | Predictor | Self-reports | *Duration of hearing loss, right ear*  0-9 years  10-19 years  20-29 years  30-39 years  40-49 years  >= 50 years  Not asked | Q: If yes, how long has it been since the hearing problems were determined for the first time (right ear)? | 101 (23.1%)  222 (50.7%)  22 (5.0%)  12 (2.7%)  4 (0.9%)  5 (1.1%)  72 (16.4%) | 162 |
| 36 | Predictor | Self-reports | *Subjective hearing problems in quiet*  Very slight  Slight  Medium  Strong  Very strong  Not asked | Q: How pronounced are your hearing problems in a quiet environment? (for HAU: without hearing aid) | 43 (9.8%)  98 (22.4%)  170 (38.8%)  43 (9.8%)  12 (2.7%)  72 (16.4%) | 2 |
| 37 | Predictor | Self-reports | *Subjective hearing problems in noise*  Very slight  Slight  Medium  Strong  Very strong  Not asked | Q: How pronounced are your hearing problems in a noisy environment? (for HAU: without hearing aid) | 14 (3.2%)  29 (6.6%)  115 (26.3%)  131 (29.9%)  77 (17.6%)  72 (16.4%) | 2 |
| 38 | Predictor | Self-reports | *Exposed to noise in job*  No  <5h/week  6-15h/week  16-30h/week  >30h/week  Not asked | Q: Have you ever been or are you currently exposed to noise in your job? | 225 (51.4%)  40 (9.1%)  42 (9.6%)  33 (7.5%)  26 (5.9%)  72 (16.4%) | 3 |
| 39 | Predictor | Self-reports | *Cause of hearing loss known*  No  Yes  Not asked | Q: Do you know the reason for your hearing impairment? | 132 (30.1%)  234 (53.4%)  72 (16.4%) | 0 |
| 40 | Predictor | Self-reports | *Sudden hearing loss*  No  Yes | Q: Have you ever had a sudden hearing loss? | 332 (75.8%)  106 (24.2%) | 1 |
| 41 | Predictor | Self-reports | *Ear noise*  No  Yes | Q: Do you have noise in ear (tinnitus)? | 222 (50.7%)  216 (49.3%) | 2 |
| 42 | Predictor | Cognitive | *Number transcoding (DemTect)*  0 points  1 points  2 points  3 points  4 points | DemTect subtest Number Transcoding | 6 (1.4%)  13 (3.0%)  28 (6.4%)  101 (23.1%)  290 (66.2%) | 0 |
| 43 | Predictor | Cognitive | *Digit reverse (DemTect)*  2 points  3 points  4 points  5 points  6 points | DemTect subtest Digit Span Reverse | 4 (0.9%)  65 (14.8%)  136 (31.1%)  153 (34.9%)  80 (18.3%) | 0 |
| 44 | Predictor | Economic-technical | *Technology commitment*  1 points  2 points  3 points  4 points  5 points | Technology Commitment mean score  Low commitment  ...  High commitment | 1 (0.2%)  21 (4.8%)  169 (38.6%)  200 (45.7%)  47 (10.7%) | 0 |
| 45 | Predictor | Grouping | *Grouping variable (Bisgaard)*  N1  N2  N3  N4  N5  S1  S2  S3 | Very mild and flat/moderately sloping  Mild and flat/moderately sloping  Moderate and flat/moderately sloping  Moderate/severe and flat/moderately sloping  Severe and flat/moderately sloping  Very mild and steep sloping  Mild and steep sloping  Moderate/severe and steep sloping | 92 (21.0)  106 (24.2)  96 (21.9)  24 (5.5)  4 (0.9)  63 (14.4)  36 (8.2)  17 (3.9) | 0 |

Note: Because of rounding percentages may add up to more or less than 100%
